# Functional Differences Between EBV- and CMV-Specific CD8^+^ T cells Demonstrate Heterogeneity of T cell Dysfunction in CLL

**DOI:** 10.1097/HS9.0000000000000337

**Published:** 2020-02-13

**Authors:** Tom Hofland, Iris de Weerdt, Sanne Endstra, Aldo Jongejan, Laura Platenkamp, Ester B.M. Remmerswaal, Perry D. Moerland, Ineke J.M. ten Berge, Mark-David Levin, Arnon P. Kater, Sanne H. Tonino

**Affiliations:** 1 Amsterdam UMC, University of Amsterdam, Department of Experimental Immunology; 2 Amsterdam UMC, University of Amsterdam, Department of Hematology, Amsterdam Infection and Immunity Institute, Cancer Center Amsterdam; 3 Amsterdam UMC, University of Amsterdam, Department of Clinical Epidemiology, Biostatistics and Bioinformatics; 4 Amsterdam UMC, University of Amsterdam, Renal Transplant Unit, Amsterdam Infection and Immunity Institute, Meibergdreef 9, Amsterdam; 5 Department of Internal Medicine, Albert Schweitzer Hospital, Dordrecht; 6 Lymphoma and Myeloma Center Amsterdam, LYMMCARE, Amsterdam, the Netherlands.

## Abstract

Acquired T cell dysfunction is a hallmark of chronic lymphocytic leukemia (CLL), and is linked to an increased risk of infections, but also reduced immune surveillance and disappointing responses to autologous T cell-based immunotherapy. The mechanisms of T cell dysfunction in CLL are not well understood. Studying immunity against chronic viruses allows for detailed analysis of the effect of CLL on T cells chronically exposed to a specific antigen. Cytomegalovirus (CMV) reactivations are rare in CLL, which corroborates with preserved CMV-specific T cell function. Epstein-Barr virus (EBV) is another herpesvirus that results in chronic infection, but unlike CMV, is characterized by subclinical reactivations in CLL patients. Since both herpesviruses induce strong CD8^+^ T cell responses, but have different clinical outcomes, studying these specific T cells may shed light on the mechanisms of CLL-induced T cell dysfunction.

We first analyzed the phenotype of EBV-specific CD8^+^ T cells in CLL and healthy controls, and found that in CLL EBV-specific CD8^+^ T cells are in an advanced differentiation state with higher expression of inhibitory receptors. Secondly, CLL-derived EBV-specific CD8^+^ T cells show reduced cytotoxic potential, in contrast to CMV-specific T cells. Finally, we performed transcriptome analysis to visualize differential modulation by CLL of these T cell subsets. While T cell activation and differentiation genes are unaffected, in EBV-specific T cells expression of genes involved in synapse formation and T cell exhaustion is altered. Our findings on the heterogeneity of antigen specific T cell function in CLL aids in understanding immune-dysregulation in this disease.

## Introduction

CLL is characterized by an acquired dysfunction of the T cell compartment, which results in an increased risk of infections and possibly decreased antitumor immunity.^1,2^ The acquired T cell dysfunction is generally also considered to be responsible for the hampered activity of autologous T cell mediated therapies in CLL.^3,4^ Understanding the biology of this acquired T cell dysfunction is an important aspect of the search for means to restore T cell function in CLL. T cells from CLL patients show an increased expression of inhibitory receptors (e.g. PD-1, CD160 and CD244), reduced proliferative capacity, limited cytotoxicity and impaired immune synapse formation.^5,6^ Most studies so far have focused on the effects of CLL on the T cell compartment as a whole. Although CLL has been shown to induce transcriptional changes in both the global CD4^+^ and CD8^+^ T cell compartments, the profound skewing of T cell differentiation states in CLL might obscure differences in specific T cell subsets between CLL patients and healthy controls (HC).^7^ Studying well defined T cell responses to specific antigens within the CLL environment may provide detailed insight in how CLL influences T cell function.

Cytomegalovirus (CMV) reactivations are common during various situations of reduced T cell function (eg, after allo-HSCT), but exceedingly rare in untreated CLL patients, despite the reported T cell defects. We have previously demonstrated that CMV-specific CD8^+^ T cells are fully functional in CLL.^8^ This indicates that T cell function in CLL is more heterogeneously affected than previously assumed, with at least one subset of T cells able to escape tumor-induced dysfunction.

Epstein-Barr virus (EBV) is another herpesvirus that results in chronic latent infection, and has a high prevalence (>90%) in the adult population.^9^ In healthy individuals, CD8^+^ T cells are responsible for immunological control of EBV.^9–11^ Although clinical reactivations of EBV in CLL patients are rare, several studies have shown an increased frequency of subclinical reactivations of EBV in CLL patients. In some studies, these reactivations correlated with shorter time-to-first-treatment and reduced overall survival.^12–17^ The increased frequency of EBV reactivations may indicate a decreased function of EBV-specific CD8^+^ T cells in CLL patients.

The differences in clinical reactivations imply distinct immune responses towards these related herpesviruses in CLL. Comparing EBV- and CMV-specific T cells in CLL may serve as a tool to understand T cell modulation by CLL, and complement earlier studies in which global T cell compartments of CLL and HC were compared. Here, we studied the phenotype, function and transcriptome of EBV and CMV-specific CD8^+^ T cells of untreated CLL patients and age-matched HC.

## Results

### EBV-specific CD8^+^ T cells of CLL patients show impaired cytotoxicity

**Figure 1:**
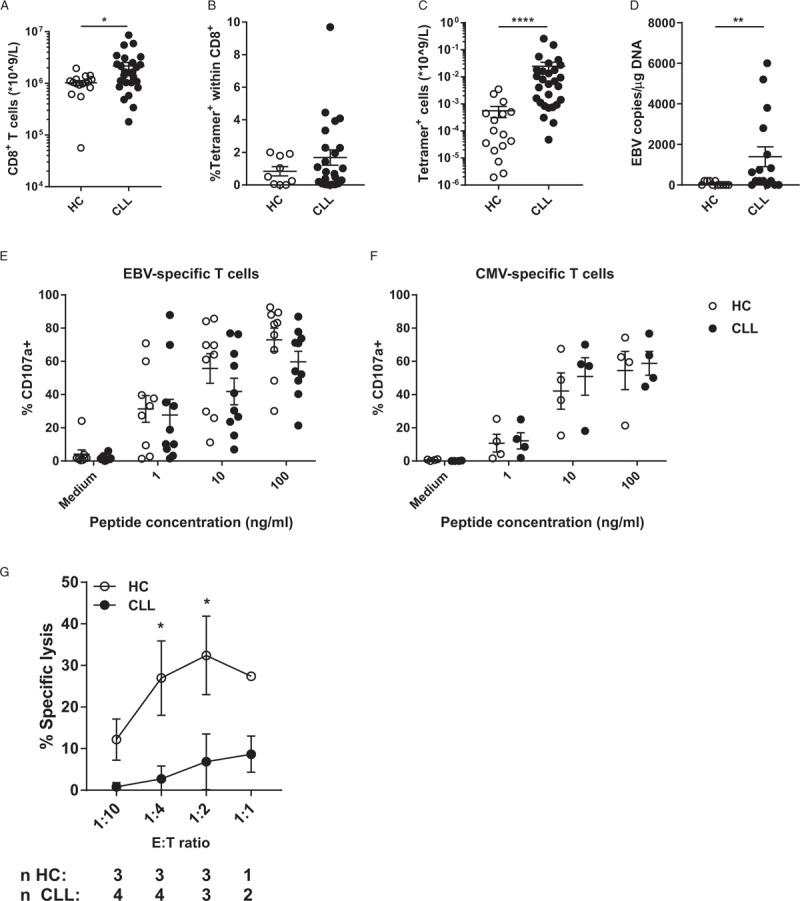
**EBV-specific CD8**^**+**^** T cells are expanded but lack cytotoxic function in CLL**. EBV-specific CD8^+^ T cells of HC (n = 16) and CLL patients (n = 27) were identified by tetramer staining. (A) Absolute numbers of global CD8^+^ T cells, (B) relative frequency of EBV-specific CD8^+^ T cells within the CD8^+^ T cell population and (C) absolute number of EBV-specific CD8^+^ T cells in peripheral blood. (D) EBV viral load, measured as copies of EBV DNA within isolated DNA from PBMC by PCR. (E) Degranulation of EBV-specific CD8^+^ T cells after EBV peptide stimulation for 5 hours, measured by CD107a^+^ cells by flow cytometry (n = 10 for CLL, n = 9 for HC). (F) Degranulation of CMV-specific CD8^+^ T cells after CMV peptide stimulation for 5 hours (n = 4 for CLL and HC). (G) Cytotoxicity of EBV-specific CD8^+^ T cells from CLL patients and HC. Allogeneic B cells were labeled with CFSE and loaded overnight with EBV peptides (10 μg/ml). The next day, enriched CD8^+^ T cells derived from CLL patients (n = 4) or HC (n = 3) were co-cultured with CFSE-labeled allogeneic B-cells for 16–18 hours. Target cell death was determined by staining with MitoTracker Orange and To-pro-3. ^∗^ = p < 0.05; ^∗∗^ = p < 0.01; ^∗∗∗∗^ = p < 0.0001 (Mann-Whitney *U* test and two-way repeated measures ANOVA with Sidak's multiple comparisons test).

We analyzed EBV-specific CD8^+^ T cell numbers in both CLL patients and age-matched HC using virus-specific tetramers (gating strategies in Supplementary Figure 1). In accordance with earlier reports, we found an increase in total CD8^+^ T cell numbers in CLL (Fig. 1A). The relative frequency of EBV-specific CD8^+^ T cells within the global CD8^+^ T cell pool is not changed in CLL (Fig. 1B), but due to the increased absolute number of CD8^+^ T cells we observed an increase in the absolute number of EBV-specific CD8^+^ T cells in CLL patients (Fig. 1C). These results indicate the expansion of CD8^+^ T cell subsets is not outcompeting EBV-specific CD8^+^ T cells in CLL. Despite the presence of EBV-specific CD8^+^ T cells, CLL patients have significantly higher EBV viral loads compared to HC, which may indicate functional impairment of EBV-specific CD8^+^ T cells in CLL (Fig. 1D). As defects in cytotoxic function have been reported for CD8^+^ T cells in CLL, we measured the cytotoxic potential of EBV-specific CD8^+^ T cells in CLL patients. We observed a trend towards decreased degranulation of EBV-specific CD8^+^ T cells of CLL patients after EBV peptide stimulation (Fig. 1E). In contrast, CMV-specific CD8^+^ T cells showed similar levels of degranulation in CLL compared to HC, in line with our earlier report on their functional cytotoxicity (Fig. 1F).^8^ Degranulation of EBV-specific CD8^+^ T cells is not impaired after PMA/Ionomycin stimulation, demonstrating there is no intrinsic functional defect in degranulation of EBV-specific CD8^+^ T cells in CLL (Supplementary Fig. 2A). To study whether the decrease in degranulation translated into defective cytotoxicity, enriched CD8^+^ T cells from both CLL patients and HC were co-cultured with EBV-peptide loaded third-party B-cells and target cell death was analyzed by flow cytometry (Supplemental Fig. 2B+C). Although only few donors exhibited enough EBV-specific CD8^+^ T cells to conduct the assay, CLL-derived EBV-specific CD8^+^ T cells showed a significantly decreased ability to induce target cell death compared to HC (Fig. 1G). This shows that EBV-specific CD8^+^ T cells from CLL patients, in contrast to the CMV-specific CD8^+^ T cell compartment, have decreased cytotoxic function.^8^

### EBV-specific T cells of CLL patients display an advanced differentiation state

**Figure 2:**
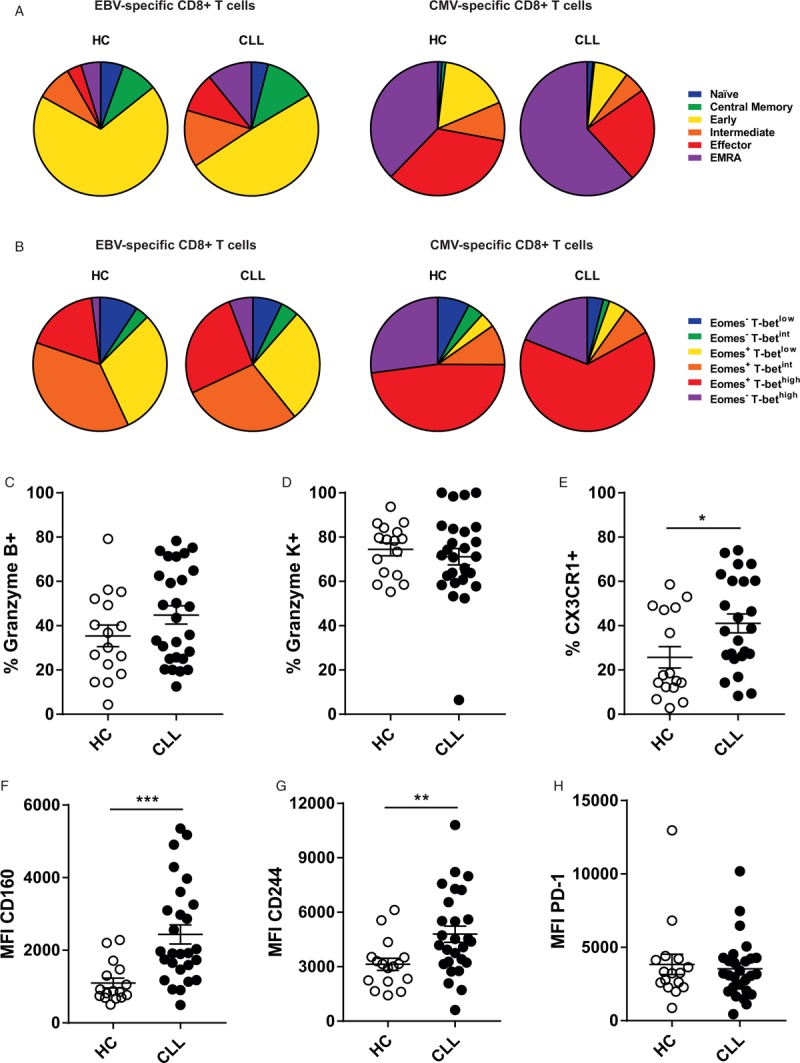
EBV-specific CD8^+^ T cells show an increased differentiation state and an increased expression of exhaustion markers in CLL patients. Phenotypic markers of T cell differentiation, effector function and inhibitory receptors on virus-specific CD8^+^ T cell populations of HC (n = 16) and CLL patients (n = 27). (A) Differentiation state of EBV- and CMV-specific CD8^+^ T cells based on extracellular surface markers (naïve: CD45RA+CD27+CCR7+CD28+; central memory: CD45RA^-^CCR7^+^CD27^+^CD28^+^; early: CD45RA^-^CCR7^-^ CD27^+^CD28^+^; intermediate: CD45RA^-^CCR7^-^CD27^-^CD28^+^; effector: CD45RA^-^CCR7^-^CD27^-^CD28^-^; EMRA: CD45RA^+^CCR7^-^CD27^-^CD28^-^). (B) Differentiation state of EBV- and CMV-specific CD8^+^ T cells based on transcription factors T-bet and Eomes. (C-H) Expression levels of effector molecules (C) Granzyme B, (D) Granzyme K, (E) CX3CR1, (F) CD160, (G) CD244 and (H) PD-1 by EBV-specific CD8^+^ T cells. ^∗^ = p < 0.05 ^∗∗^ = p < 0.01; ^∗∗∗^ = p < 0.001 (one-way ANOVA with Bonferroni correction, Students *t* test, Mann-Whitney *U* test).

To gain more insight in the underlying mechanism of reduced cytotoxicity, we analyzed the effector potential of EBV-specific CD8^+^ T cells in CLL. Based on expression of extracellular surface markers (CD45RA, CCR7, CD27 and CD28), EBV-specific CD8^+^ T cells of CLL patients displayed an advanced differentiation state compared to HC-derived T cells, with a significant decrease in cells with an early effector memory phenotype and an increase in more differentiated effector populations (Fig. 2A). This parallels the global CD8^+^ T cell compartment, in which significant decreases in naïve and early effector memory cells, and a significant increase in effector cells are seen (Supplemental Fig. 3A+C). In contrast to the EBV-specific compartment, the CMV-specific compartment in HC largely consists of highly differentiated effector cells. In CLL, we also observed a skewing towards effector populations in CMV-specific CD8^+^ T cells, with a relative increase in Temra cells (Fig. 2A), as was reported earlier.^18^ However, as CMV-specific CD8^+^ T cells in HC are already highly differentiated, the shift seen in CLL is relatively small compared to the changes in the EBV-specific compartment. The phenotypic results were confirmed by the expression of two master regulators of CD8^+^ T cell differentiation, T-bet and Eomes.^19–24^ EBV- and CMV-specific CD8^+^ T cells of CLL patients showed higher expression levels of the transcription factor T-bet, which drives CD8^+^ T cell effector function (Fig. 2B, global CD8^+^ T cells in Supplemental Figure 3B). The increased differentiation of EBV-specific CD8^+^ T cells in CLL patients is accompanied by trends towards higher expression of granzyme B, lower expression of granzyme K, and a significant increase in expression of the fractalkine receptor CX3CR1 (Fig. 2C–E, global CD8^+^ T cells in Supplemental Fig. 3D–F). These phenotypic data all corroborate a skewing towards effector differentiation of EBV-specific CD8^+^ T cells in CLL.

### EBV-specific T cells of CLL patients have higher expression of inhibitory receptors

We measured the expression levels of several key inhibitory receptors which were reported to be overexpressed on the global CD8^+^ T cell compartment in CLL, but not on CMV-specific CD8^+^ T cells.^6,8^ CLL-derived EBV-specific CD8^+^ T cells have an increased expression of CD160 and CD244 compared to HC, however, the expression of PD-1 is not significantly altered (Fig. 2F–H). Similar patterns were seen in the global CD8^+^ T cell pool (Supplemental Fig. 3F–H), which could indicate that EBV-specific CD8^+^ T cells are a representative subset to study immunomodulatory effects of CLL in more detail.

### Cytokine production by EBV-specific CD8^+^ T cells of CLL patients is unaffected

**Figure 3:**
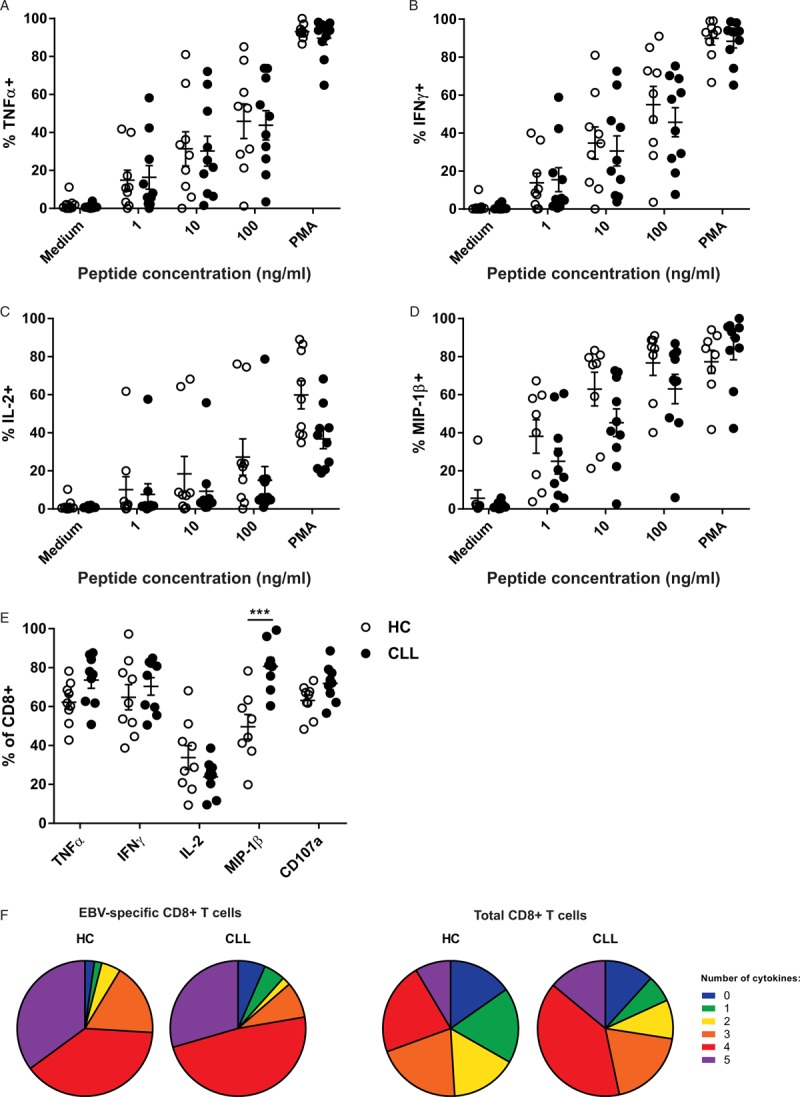
**EBV-specific CD8**^**+**^** T cells of CLL patients display normal cytokine production**. EBV-specific CD8^+^ T cells derived from CLL patients (n = 10) or HC (n = 9) were stimulated for 5 hours with varying concentrations of EBV peptides (1–100 ng/ml) or with PMA/Ionomycin. Cytokine production by EBV-specific CD8^+^ T cells was analyzed by flow cytometry. (A–E) Production of (A) TNFα, (B) IFNγ, (C) IL-2, (D) MIP-1β by EBV-specific CD8^+^ T cells after stimulation. (E) Production of cytokines and degranulation by the total CD8^+^ T cell pool after PMA stimulation. (F) Polyfunctionality of EBV-specific CD8^+^ T cells and the total CD8^+^ T cell pool after PMA stimulation for both CLL patients and HC (also including degranulation). ^∗∗∗^ = p < 0.001 (two-way repeated measures ANOVA with Sidak's multiple comparisons test).

Next, we analyzed whether EBV-specific CD8^+^ T cells of CLL patients retain the capability to respond to peptide stimulation, despite the elevated expression of inhibitory receptors. We analyzed the capability of the EBV-specific CD8^+^ T cell pool to produce effector cytokines by stimulating T cells with EBV peptides. As a positive control, stimulation with PMA/Ionomycin was used (gating strategy in Supplemental Fig. 4). We found that EBV-specific CD8^+^ T cells of CLL patients produce similar levels of the classical CD8^+^ T cell effector cytokines TNFα, IFNγ, IL-2, and MIP-1β as HC after stimulation (Fig. 3A–D). In the global CD8^+^ T cell pool, we observed a trend towards an increased production of TNFα, IFNγ, a significant increase of MIP-1β production, and a trend towards lower IL-2 production after PMA/Ionomycin stimulation (Fig. 3E). These results are similar to those reported by others.^6,8^ There were no differences in the polyfunctionality in either the EBV-specific or total CD8^+^ T cell pool between CLL patients and HC, indicating that cytokine production by EBV-specific CD8^+^ T cells is functionally intact in CLL patients, and is not implicated in T cell dysfunction in CLL (Fig. 3F).

### RNA sequencing reveals differential alteration of EBV- and CMV-specific CD8^+^ T cells in CLL patients

**Figure 4:**
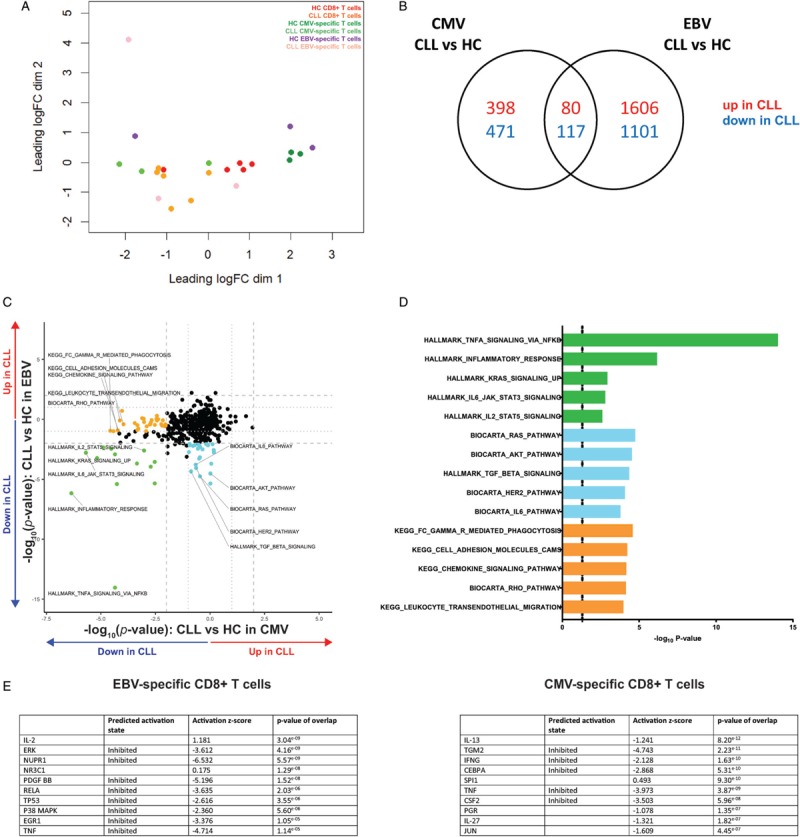
**Transcriptome analysis of EBV- and CMV-specific CD8**^**+**^** T cells shows alteration of different genesets in CLL**. Transcriptome analysis was performed on EBV- and CMV-specific CD8^+^ T cells of CLL patients (n = 3) and HC (n = 3) with RNAseq. (A) Multi-dimensional scaling plot of total, EBV- and CMV-specific CD8^+^ T cell populations. (B) Venn diagram of genes significantly changed in CLL EBV- or CMV-specific CD8^+^ T cells compared to their HC counterpart. Genes were considered significantly differentially expressed when their Benjamini-Hochberg corrected p value was < 0.25 and the absolute log_2_-fold-change > 1. (C) Plot showing a selection of genesets enriched in the comparisons of EBV- (y-axis) and CMV-specific (x-axis) CD8^+^ T cells between CLL vs HC. Each dot marks a geneset. Hallmark genesets and KEGG and Biocarta pathways were included into the analysis, in addition to six custom-made genesets. Genesets were determined to be significantly enriched when p value < 0.01 (dashed lines). (D) Selection of enriched genesets within EBV- and CMV-specific CD8^+^ T cells (green), uniquely in EBV-specific CD8^+^ T cells (blue) or uniquely in CMV-specific CD8^+^ T cells (orange) in CLL vs HC. Colors of bars match the colors of the scatterplot in panel C. Dashed line indicates p value of 0.05. Plotted p values correspond to the CLL vs HC comparison within the EBV-specific T cells for green and blue bars, and to the CLL vs HC comparison within the CMV-specific T cells for orange bars. Complete list of enriched genesets in each group can be found in Supplementary Table 1. (E) Upstream regulators predicted with Ingenuity Pathway Analysis to be involved as drivers of transcriptomic profile changes in EBV- and CMV-specific CD8^+^ T cells in CLL vs HC. Shown are the top 10 regulators with the highest p-value of overlap within each comparison. Activation z-score corresponds to the value based on the comparison of CLL vs HC within the specified group of virus-specific CD8^+^ T cells.

To visualize the impact of CLL on CD8^+^ T cells, and EBV- and CMV-specific populations in particular, we performed transcriptome analysis by RNAseq. Multi-dimensional scaling revealed that global, EBV- and CMV-specific CLL-derived CD8^+^ T cell populations mostly group separately from their respective HC-derived counterpart (Fig. 4A). However, despite the fact that CLL affects gene expression in both virus-specific populations, the number of significantly altered genes that overlap and change in the same direction between CMV- and EBV-specific CD8^+^ T cells is relatively low (197 genes), demonstrating that these virus-specific populations are differentially affected in CLL (Fig. 4B). Furthermore, we found that in EBV-specific CD8^+^ T cells in CLL more genes are differentially expressed (2707 genes) than in CMV-specific CD8^+^ T cells (869 genes, Fig. 4B). To analyze which pathways may account for the observed changes between EBV- and CMV-specific CD8^+^ T cells, we performed geneset enrichment analysis (list of genesets in Supplementary Table 1). Some genesets were similarly affected in both EBV- and CMV-specific CD8^+^ T cells (Fig. 4C+D, green). These were mostly related to T cell activating signaling pathways like TNFα, IL-2, and IL-6 signaling. Both CLL-derived EBV- and CMV-specific CD8^+^ T cells showed enrichment of genesets that were not significantly enriched in the other group, as can be expected based on the functional differences. For EBV-specific CD8^+^ T cells, these genesets relate to activating signaling pathways like RAS, AKT, and IL-6 signaling, but also the inhibitory TGF-β signaling pathway (Fig. 4C+D, blue). The significantly enriched genesets that were unique for the CMV-specific CD8^+^ T cells map to pathways of a different nature, mostly related to cell migration and adhesion (Fig. 4C+D, orange). We aimed to identify upstream regulators that could explain the transcriptional changes in CLL-derived CD8^+^ T cells by performing Ingenuity Pathway Analysis (IPA). For EBV-specific CD8^+^ T cells, several key T cell related signaling molecules like IL-2, ERK, platelet-derived growth factor (PDGF), Rel-A and TNF are predicted to be functionally less active in CLL (Fig. 4E). In CMV-specific CD8^+^ T cells, other upstream regulators are predicted to be less active in CLL, especially IL-13, transglutamase-2 (TGM2) and IFNγ (Fig. 4E). These results indicate that also on the transcriptome level EBV- and CMV-specific CD8^+^ T cells are differentially altered in CLL, which relates back to different upstream regulators involved in T cell function.

### T cell functional pathways are differentially affected in EBV- and CMV-specific CD8^+^ T cells in CLL

**Figure 5:**
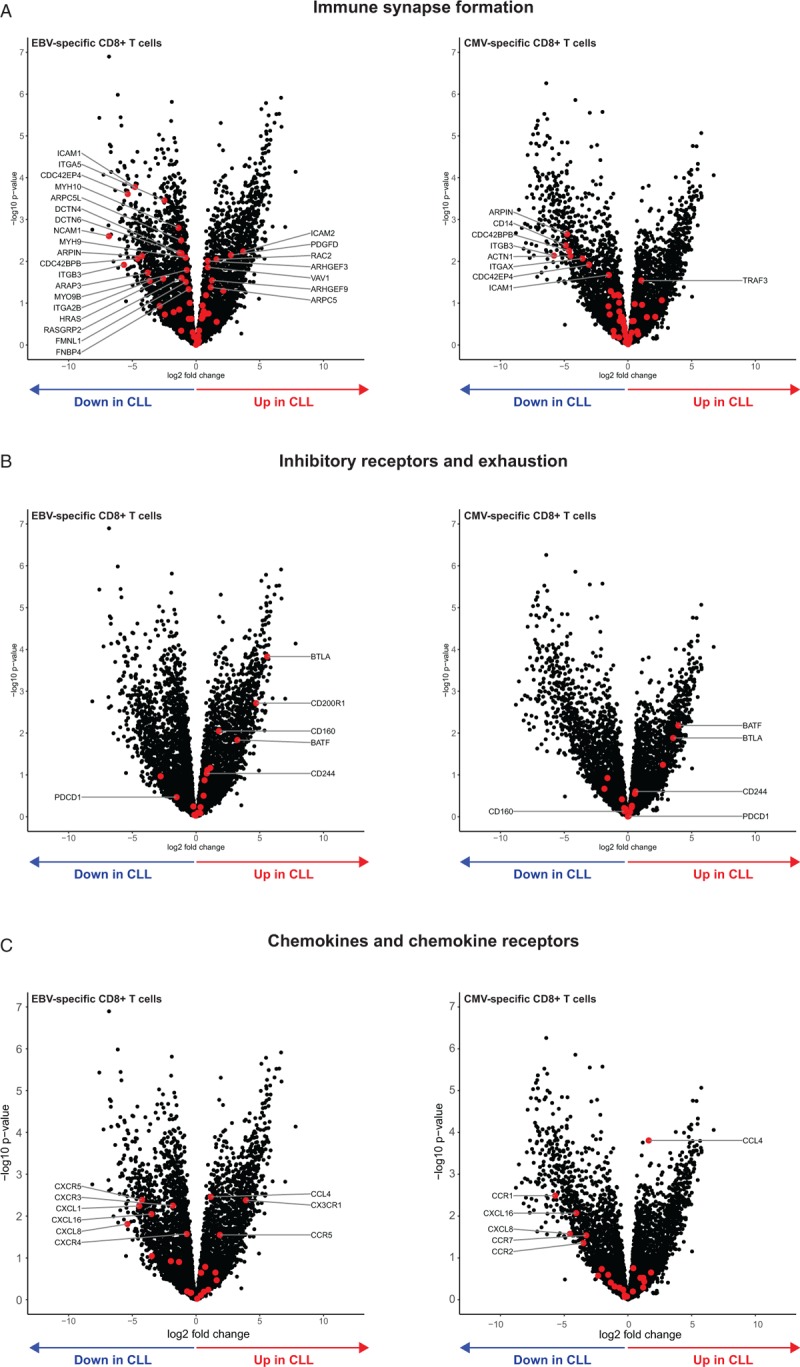
**T cell functional pathways are differentially affected in EBV- and CMV-specific CD8**^**+**^** T cells in CLL**. Volcano plots showing changes in expression of genes involved in T cell functional processes that are known to be disturbed in CLL-derived T cells. (A) Genes involved in immune synapse formation and cell adhesion. (B) Genes involved in immune inhibition and T cell exhaustion. (C) Genes involved in cellular trafficking. Left side plots show the EBV-specific CD8^+^ T cell comparison between CLL and HC, plots on the right side show comparison for CMV-specific CD8^+^ T cells. List of genes in each set can be found in Supplementary Table 3.

To investigate how transcriptional changes in CD8^+^ T cells may lead to functional impairment, we analyzed genesets involved in different stages of T cell activation and effector function. We observe that not all functional processes are affected in CLL-derived CD8^+^ T cell populations. Genes involved in early stages of T cell activation, like TCR signaling and co-stimulation, hardly show any significant differences between HC and CLL in both (dysfunctional) EBV-specific and (functional) CMV-specific CD8^+^ T cells (Supplemental Fig. 5A+B). The fact that TCR signaling appears to be unaffected fits with the finding that CD8^+^ T cells, despite reduced cytolytic capacity, are able to differentiate into effector cells and produce effector cytokines in response to EBV peptides in CLL (Figs. 2 and 3). Indeed, we observe transcriptional changes related to effector function and differentiation in EBV- and CMV-specific CD8^+^ T cells, for example IFNγ, KLRG1 and CX3CR1 (Supplemental Fig. 5C). In contrast, later stages of T cell responses are differentially affected in EBV- vs CMV-specific CD8^+^ T cells in CLL. More genes involved in immune synapse formation, already linked to reduced function of CD8^+^ T cells in CLL,^5^ show altered expression in EBV-specific CD8^+^ T cells than in CMV-specific CD8^+^ T cells in CLL (Fig. 5A). Similarly, more genes encoding inhibitory receptors and other molecules involved in T cell exhaustion show increased expression levels in the EBV-specific CD8^+^ T cell compartment in CLL (in accordance with flow cytometry data, Fig. 2F–H), compared to the CMV-specific CD8^+^ T cell fraction (Fig. 5B). Finally, the expression of genes involved in T cell trafficking was also differentially altered in EBV-specific CD8^+^ T cells compared to the CMV-specific CD8^+^ T cell compartment (Fig. 5C), as suggested by our initial geneset analysis (Fig. 4C+D). These results demonstrate that immunomodulation in CLL does not affect TCR signaling or effector differentiation, but mainly cytotoxic responses and cellular trafficking of EBV-specific CD8^+^ T cells. CMV-specific CD8^+^ T cells are less or differentially affected in these pathways and retain their functionality.

## Discussion

The mechanisms behind CLL-induced T cell dysfunction are not well understood. Studying the global CD8^+^ T cell compartment may obscure the immunomodulatory effects of CLL, as the T cell compartment is highly skewed in CLL patients. To gain insight into how CLL affects antigen-specific responses, we chose to study homogenous virus-specific T cell populations, using virus-specific CD8^+^ T cell populations directed against EBV and CMV. Both EBV and CMV induce strong CD8^+^ T cell responses, but whereas control of CMV is unaffected, CLL patients experience an increased frequency of subclinical reactivations.^23–28^

Comparison of these virus-specific CD8^+^ T cell populations by combined flowcytometric, functional, and transcriptional analyses revealed that EBV-specific CD8^+^ T cells in CLL are functionally impaired on the cytotoxic level, despite increased differentiation and expression of cytotoxic mediators. This might be explained by increased expression of inhibitory receptors and dysregulation of genes involved in immune synapse formation. Indeed, these processes were not affected in CMV-specific CD8^+^ T cells in CLL. Only a small overlap of significantly altered genes in CMV- and EBV-specific CD8^+^ T cells was found. These results demonstrate that CD8^+^ T cell subsets in CLL can be differentially affected within the same CLL micro-environment.

To what extent our findings in EBV-specific T cells apply to other T cell subsets, such as tumor-specific T cells, is unsure. EBV-specific CD8^+^ T cells seem to be in a similar “pseudo-exhausted” state as has been reported for the global CD8^+^ T cell compartment in CLL. EBV-specific CD8^+^ T cells, as well as the global CD8^+^ T cell compartment, display impaired cytotoxicity but adequate cytokine production.^5,6^ Inhibitory receptors have been shown to influence the ability to form immune synapses and cytotoxicity of CLL-derived T cells in vitro.^25^ Despite elevated expression of inhibitory receptors, treatment of CLL patients with immune checkpoint blockade therapy does not lead to clinical responses, unless patients develop Richter's transformation.^26,27^ Perhaps targeting other inhibitory receptors, or multiple receptors simultaneously, may lead to improved responses to checkpoint blockade therapy in CLL. However, since we show that CLL-derived T cells are not in a fully exhausted state, these inhibitory receptors may not play a similar important role in CLL, as in other hematologic malignancies.^27–29^ Classical T cell exhaustion requires chronic exposure to antigen in order to develop. Since CLL cells are known to have low mutational loads, CLL cells may not properly present neoantigens chronically in order for T cells to develop a classical exhausted phenotype.^30,31^ But, since CLL-neoantigen specific T cells are difficult to identify, quantitative and qualitative analyses of CLL-specific CD8^+^ T cell responses are lacking.^31,32^

As the role of T cell exhaustion in CLL remains unclear, unraveling the antigen-independent effects of CLL on T cell function may be an important aspect to study further. Indeed, the data we present here on changes in virus-specific CD8^+^ T cells are induced by CLL in a tumor antigen-independent manner. Our dataset may provide insight on qualities CD8^+^ T cells require in order to retain their functionality within the CLL micro-environment. For example, the observation of downregulation of IL-6 pathways and increased differentiation in dysfunctional EBV-specific CD8^+^ T cells match with data of an earlier study that found reduced IL-6 signatures and increased differentiation of CAR T cells in CLL patients not responding to CAR therapy.^33^

Our data indicate that several aspects of CD8^+^ T cell function are not modulated by CLL. Transcriptome analysis indicates that dysfunctional T cells are not affected by CLL with regard to TCR signaling and co-stimulatory pathways, which is in line with adequate effector differentiation and cytokine production of CD8^+^ T cells. This indicates that CLL mainly affects effector responses of T cells, in particular those associated with cytotoxicity and synapse formation. Although CD8^+^ T cell dysfunction is mainly attributed to decreased cytotoxicity, it is still unclear how CLL cells interfere in cytotoxic responses of T cells, and how CMV-specific CD8^+^ T cells escape this CLL-induced impairment. It could be that the sensitivity of CD8^+^ T cells to the induction of cytotoxic dysfunction by CLL cells is dependent on their differentiation state, which is different for EBV- and CMV-specific CD8^+^ T cells, before it comes in contact with the CLL environment.^34,35^ CMV infection results in highly differentiated CD8^+^ T cells with a high cytotoxic potential, that expand and persist for long periods of time.^36^ EBV-specific CD8^+^ T cells have a less differentiated phenotype, and the fact that these cells require additional differentiation to obtain full cytotoxic potential could explain their susceptibility for CLL-induced immunomodulation.

In conclusion, our results indicate that, unlike T cells that recognize CMV, EBV-specific CD8^+^ T cells are not able to escape CLL-induced T cell dysfunction. We thereby demonstrate that T cell subsets are differentially altered by the CLL micro-environment, indicating that CLL-induced T cell dysfunction is more heterogeneous than was previously assumed. Our results provide in depth profiles of CD8^+^ T cells with different functional capabilities within the CLL micro-environment. Further studies into how specific T cell subsets are able to remain functional within the CLL micro-environment can shed more light on the molecular determinants of T cell dysfunction in CLL, and may result in new possibilities to enhance the efficacy of T cell mediated immunotherapy in CLL.

## Materials and methods

### Patient and healthy donor samples

Peripheral blood samples from untreated CLL patients were collected at the Amsterdam University Medical Centers, location AMC, and the Albert Schweitzer Hospital. Age-matched healthy controls (HC) served as the control group: all had normal lymphocyte counts and monoclonal B-cell lymphocytosis was excluded by CD19, CD5, κ, and λ immunophenotyping (<5% of lymphocytes for all donors). Healthy controls were recruited at the University Medical Centers, location AMC, or obtained from Sanquin Blood Supply, Amsterdam. All CLL and control samples used in this study tested positive for EBV serology and expression of HLA-A2, -A3, -B7 and/or –B8. For a brief overview of CLL patient and HC characteristics, and data on CMV serology, see Table 1. Ethical approval was given by the medical ethical committee at the University Medical Centers, location AMC, and written informed consent of both patients and healthy donors was obtained in accordance with the Declaration of Helsinki.

**Table 1:**
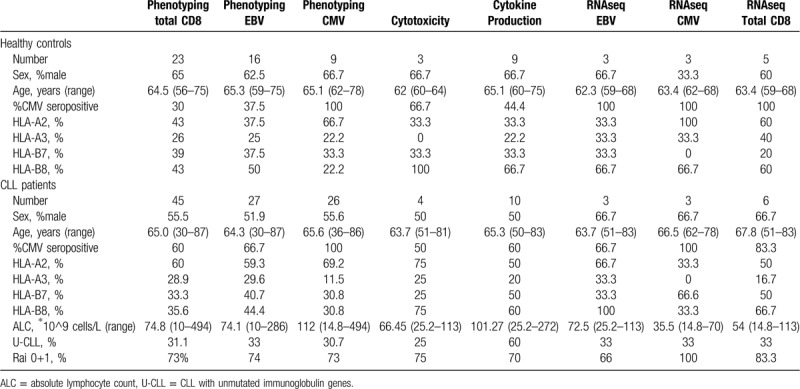
Characteristics of Patients and Healthy Controls.

### Flow cytometry analysis of EBV-specific T cells

Thawed peripheral blood mononuclear cells (PBMC) were stained with EBV- or CMV-specific tetramers, followed by extracellular and intracellular FACS antibodies. An overview of tetramers and FACS antibodies used in this study is provided in supplementary table 2. Cells were analyzed on an LSRFortessa flow cytometer (BD Biosciences). Data analysis was performed using Flowjo Mac Version 10.

### Cytotoxicity of EBV-specific T cells

CD8^+^ T cells were enriched by positive immunomagnetic microbead isolation (Miltenyi Biotec). Enriched CD8^+^ T cells were stained with tetramers to determine EBV-specific T cell frequency. Unrelated HC CD19^+^ B-cells were isolated by CD19 MACS, labeled with carboxyfluorescein succinimidyl ester (CFSE; Thermo Fisher Scientific), and loaded with EBV peptides (10 μg/ml, Netherlands Cancer Institute, Amsterdam) overnight at 37°C. CD19^+^ target cells were derived from donors expressing the relevant HLA molecules required for antigen presentation of the selected EBV peptides. Enriched CD8^+^ T cells and CD19^+^ target cells were co-incubated overnight at 37°C at various effector:target ratio's. Afterwards, cells were stained with MitoTracker Orange (Invitrogen) for 25 minutes at 37°C, followed by staining with To-pro-3 (Invitrogen) for 15 minutes at room temperature. Live cells were defined as MitoTracker Orange^+^ and To-pro-3^-^. Cells were analyzed on a FACSCanto flow cytometer. Specific lysis of target cells was calculated as (% target cell death peptide loaded sample − % target cell death unloaded sample)/(100 – target cell death unloaded sample).

### Cytokine production by CMV and EBV-specific T cells

PBMC of CLL samples were enriched for T cells by CD19 depletion as described above. PBMC of CLL and HC were incubated with the appropriate CMV or EBV peptides at various concentrations (1–100 ng/ml) in combination with anti-CD28 (15E8, 2 μg/ml), anti-CD29 (TS 2/16, 1 μg/ml), brefeldin A (10 μg/ml, Sigma-Aldrich, St Louis, MO, USA) and GolgiStop (BD Biosciences) and incubated at 37°C for 5 hours. Then, cells were washed, stained with appropriate CMV- or EBV-specific tetramers, followed by extracellular and intracellular staining using the Cytofix/Cytoperm kit (BD Biosciences). Cells were analyzed on an LSRFortessa flow cytometer (BD Biosciences). Data analysis was performed using Flowjo Mac Version 10.

### RNA sequencing of EBV- and CMV-specific CD8^+^ T cells

EBV- and CMV-specific CD8^+^ T cells were sorted on a FACSAria cell sorter (BD Biosciences) by gating CD3^+^CD8^+^Tetramer^+^ viable lymphocytes; total CD8 cells were gated as tetramer negative CD3^+^CD8^+^. RNA isolation and sequencing was performed as described earlier.^37^ In short, RNA from sorted populations was isolated using the Nucleospin RNA Isolation kit (Machery Nagel, Düren, Germany). cDNA synthesis and library preparation were performed with Ovation RNA-seq System V2 and Ovation Ultra Low System V2 kits, respectively (Nugen, San Carlos, CA). Single-end 75 basepair sequencing was performed with an Illumina NextSeq 500 sequencer at GenomeScan (Leiden, the Netherlands). Data analysis was performed using R (v3.4.3) and Bioconductor (v3.6) (a more detailed description can be found in supplemental material). Geneset enrichment analysis was performed using CAMERA^38^ with Hallmark, KEGG, Biocarta genesets obtained from the Molecular Signature Database (MSigDB v6.1) in combination with six manually created genesets (Supplementary Table 3). Ingenuity Pathway Analysis (IPA, https://www.qiagenbioinformatics.com/products/ingenuity-pathway-analysis/) was performed using genes with a Benjamini-Hochberg corrected p value < 0.25 and an absolute log_2_-fold-change > 1 when comparing CLL vs HC either within the EBV-specific T cells or CMV-specific T cells. Sequence data has been made available in the European Genome-phenome Archive (EGA), accession number: EGAS00001003566.

### Statistical analysis

Data were analyzed using Student's *t* test, Mann-Whitney *U* test, one-way ANOVA with Bonferroni correction, or two-way repeated measures ANOVA with Sidak's multiple comparisons test, as indicated. Statistical tests were performed using GraphPad Prism 6. Differences were considered statistically significant when p values were ≤0.05. Data are presented as mean + SEM.

## Supplementary Material

Supplemental Digital Content

## Supplementary Material

Supplemental Digital Content

## Supplementary Material

Supplemental Digital Content

## Supplementary Material

Supplemental Digital Content

## Supplementary Material

Supplemental Digital Content

## Supplementary Material

Supplemental Digital Content

## Supplementary Material

Supplemental Digital Content

## Supplementary Material

Supplemental Digital Content

## Supplementary Material

Supplemental Digital Content
