# Supplementary material for: Functional Differences Between EBV- and CMV-Specific CD8+ T cells Demonstrate Heterogeneity of T cell Dysfunction in CLL
Source: Hemasphere. 2020 Feb 13;4(2):e337. doi: 10.1097/HS9.0000000000000337 (PMC7162091; doi:10.1097/HS9.0000000000000337)
Supplement: Supplemental Digital Content [file hs9-4-e337-s005.docx]

**Supplementary Table 1: Geneset enrichment analysis of Hallmark, KEGG and Biocarta pathways in EBV- and CMV-specific CD8^+^ T cells in CLL patients and HC**

| **Geneset name** | **Adj. p-value EBV CLL vs HC** | **Adj. p-value CMV CLL vs HC** |
| --- | --- | --- |
| HALLMARK_TNFA_SIGNALING_VIA_NFKB | 7.43E-12 | 0.004909 |
| HALLMARK_INFLAMMATORY_RESPONSE | 0.000132 | 0.000386 |
| HALLMARK_ANGIOGENESIS | 0.000684 | 0.004909 |
| HALLMARK_UV_RESPONSE_UP | 0.001708 | 0.062221 |
| HALLMARK_P53_PATHWAY | 0.010772 | 0.023825 |
| HALLMARK_EPITHELIAL_MESENCHYMAL_TRANSITION | 0.010772 | 0.023825 |
| HALLMARK_APOPTOSIS | 0.020116 | 0.024727 |
| HALLMARK_IL6_JAK_STAT3_SIGNALING | 0.046135 | 0.002943 |
| HALLMARK_KRAS_SIGNALING_UP | 0.06666 | 0.010336 |
| HALLMARK_HYPOXIA | 0.081312 | 0.00249 |
| KEGG_HEMATOPOIETIC_CELL_LINEAGE | 0.103059 | 0.002943 |
| HALLMARK_IL2_STAT5_SIGNALING | 0.110259 | 0.039851 |
| KEGG_ASTHMA | 0.122493 | 0.004909 |
| KEGG_CYTOKINE_CYTOKINE_RECEPTOR_INTERACTION | 0.122493 | 0.004909 |
|  |  |  |
| KEGG_SPLICEOSOME | 0.001708 | 0.835375 |
| BIOCARTA_RAS_PATHWAY | 0.00304 | 0.567011 |
| BIOCARTA_AKT_PATHWAY | 0.004849 | 0.952557 |
| HALLMARK_TGF_BETA_SIGNALING | 0.007044 | 0.508198 |
| BIOCARTA_IL6_PATHWAY | 0.010772 | 0.636014 |
| BIOCARTA_HER2_PATHWAY | 0.010772 | 0.727984 |
| BIOCARTA_ACH_PATHWAY | 0.046135 | 0.757233 |
| BIOCARTA_INSULIN_PATHWAY | 0.046135 | 0.796087 |
| KEGG_PROSTATE_CANCER | 0.047421 | 0.368088 |
| BIOCARTA_IGF1_PATHWAY | 0.049399 | 0.820072 |
| KEGG_CIRCADIAN_RHYTHM_MAMMAL | 0.051972 | 0.84873 |
| KEGG_ACUTE_MYELOID_LEUKEMIA | 0.062323 | 0.583101 |
| BIOCARTA_TNFR2_PATHWAY | 0.089446 | 0.736058 |
| BIOCARTA_UCALPAIN_PATHWAY | 0.09164 | 0.321209 |
| BIOCARTA_GSK3_PATHWAY | 0.092384 | 0.475654 |
| BIOCARTA_TFF_PATHWAY | 0.122493 | 0.454269 |
| BIOCARTA_INFLAM_PATHWAY | 0.122493 | 0.616244 |
| BIOCARTA_PROTEASOME_PATHWAY | 0.126311 | 0.593553 |
| BIOCARTA_LONGEVITY_PATHWAY | 0.128176 | 0.756432 |
|  |  |  |
| KEGG_FC_GAMMA_R_MEDIATED_PHAGOCYTOSIS | 0.493133 | 0.003927 |
| KEGG_CELL_ADHESION_MOLECULES_CAMS | 0.563639 | 0.004909 |
| KEGG_CHEMOKINE_SIGNALING_PATHWAY | 0.385849 | 0.004909 |
| KEGG_PATHOGENIC_ESCHERICHIA_COLI_INFECTION | 0.554297 | 0.005282 |
| KEGG_LEUKOCYTE_TRANSENDOTHELIAL_MIGRATION | 0.744843 | 0.006932 |
| HALLMARK_XENOBIOTIC_METABOLISM | 0.387578 | 0.007683 |
| KEGG_VIBRIO_CHOLERAE_INFECTION | 0.985736 | 0.007838 |
| BIOCARTA_MPR_PATHWAY | 0.554297 | 0.008141 |
| HALLMARK_MTORC1_SIGNALING | 0.385849 | 0.008181 |
| BIOCARTA_RHO_PATHWAY | 0.894114 | 0.012364 |
| BIOCARTA_LYM_PATHWAY | 0.679844 | 0.022669 |
| BIOCARTA_GLYCOLYSIS_PATHWAY | 0.679844 | 0.022815 |
| HALLMARK_APICAL_JUNCTION | 0.405428 | 0.023715 |
| BIOCARTA_BLYMPHOCYTE_PATHWAY | 0.719636 | 0.023825 |
| HALLMARK_FATTY_ACID_METABOLISM | 0.679844 | 0.026537 |
| BIOCARTA_MONOCYTE_PATHWAY | 0.929412 | 0.026537 |
| HALLMARK_ALLOGRAFT_REJECTION | 0.867021 | 0.029584 |
| BIOCARTA_CBL_PATHWAY | 0.744843 | 0.030279 |
| KEGG_REGULATION_OF_ACTIN_CYTOSKELETON | 0.803072 | 0.042989 |
| KEGG_GLYCEROPHOSPHOLIPID_METABOLISM | 0.65234 | 0.045528 |
| BIOCARTA_SALMONELLA_PATHWAY | 0.527201 | 0.045528 |
| HALLMARK_REACTIVE_OXIGEN_SPECIES_PATHWAY | 0.563639 | 0.048778 |
| KEGG_GLYCOLYSIS_GLUCONEOGENESIS | 0.962271 | 0.049033 |
| HALLMARK_GLYCOLYSIS | 0.667205 | 0.059212 |
| BIOCARTA_CCR3_PATHWAY | 0.694946 | 0.060601 |
| BIOCARTA_CDC42RAC_PATHWAY | 0.957812 | 0.061775 |
| BIOCARTA_ACTINY_PATHWAY | 0.73911 | 0.074255 |
| HALLMARK_OXIDATIVE_PHOSPHORYLATION | 0.944285 | 0.082048 |
