# Supplementary material for: Functional Differences Between EBV- and CMV-Specific CD8+ T cells Demonstrate Heterogeneity of T cell Dysfunction in CLL
Source: Hemasphere. 2020 Feb 13;4(2):e337. doi: 10.1097/HS9.0000000000000337 (PMC7162091; doi:10.1097/HS9.0000000000000337)
Supplement: Supplemental Digital Content [file hs9-4-e337-s006.pdf]

Supplemental Figure 5

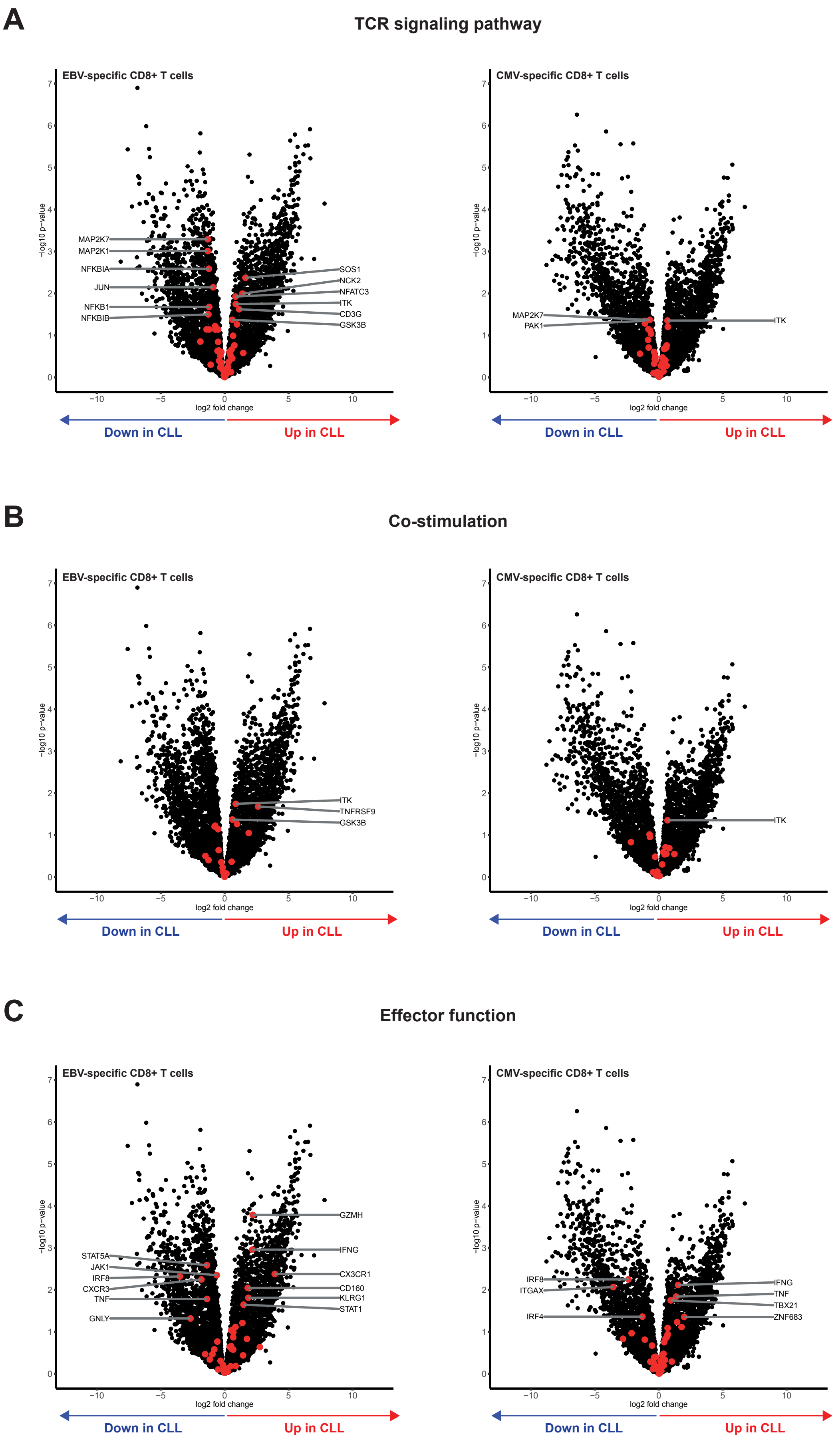

Supplemental Figure 5: Genes involved in TCR signaling and co-stimulation are not significantly altered within virus-specific CD8^{+} T cells. Volcano plots showing changes in the expression of genes involved in (A) TCR signaling (B) co-stimulation and (C) effector function within EBV- (left) and CMV-specific (right) CD8^{+} T cells.
