# Supplementary material for: Functional Differences Between EBV- and CMV-Specific CD8+ T cells Demonstrate Heterogeneity of T cell Dysfunction in CLL
Source: Hemasphere. 2020 Feb 13;4(2):e337. doi: 10.1097/HS9.0000000000000337 (PMC7162091; doi:10.1097/HS9.0000000000000337)
Supplement: Supplemental Digital Content [file hs9-4-e337-s007.pdf]

ENSG	Gene	Encodes	Effector	TCR pathway	Costimulation	Inhibitory/exhaustion	Synapse/adhesion	Chemokines/receptors
ENSG00000111537	IFNG	IFNg	x					
ENSG00000109471	IL2	IL-2	x					
ENSG00000232810	TNF	TNFa	x					
ENSG00000271503	CCL5	CCL5	x					x
ENSG00000139187	KLRG1	Klrg1	x					
ENSG00000117560	FASLG	Fas ligand	x					
ENSG00000121858	TNFSF10	TRAIL	x					
ENSG00000134545	KLRC1	Klrc1 / NKG2A	x					
ENSG00000213809	KLRK1	Klrk1 / NKG2D	x					
ENSG00000188389	PDCD1	PD-1	x			x		
ENSG00000117281	CD160	CD160	x			x		
ENSG00000134460	IL2RA	IL-2 receptor subunit alpha	x					
ENSG00000110324	IL10RA	IL-10 receptor subunit alpha	x					
ENSG00000115604	IL18R1	IL-18 receptor 1	x					
ENSG00000115607	IL18RAP	IL-18 receptor accessory protein	x					
ENSG00000081985	IL12RB2	IL-12 receptor beta 2	x					
ENSG00000186810	CXCR3	CXCR3	x					x
ENSG00000005844	ITGAL	CD11a	x					
ENSG00000160255	ITGB2	CD18; Integrin beta-2	x				x	
ENSG00000156886	ITGAD	CD11d	x					
ENSG00000140678	ITGAX; Integrin alpha-X	CD11c	x				x	
ENSG00000115232	ITGA4	CD49d; Integrin alpha-4	x				x	

ENSG00000169896	ITGAM	CD11b; Integrin alpha-M	x				x	
ENSG00000138378	STAT4	Stat4	x					
ENSG00000115415	STAT1	Stat1	x					
ENSG00000170581	STAT2	Stat2	x					
ENSG00000126561	STAT5a	Stat5a	x					
ENSG00000162434	JAK1	Jak1	x					
ENSG00000100453	GZMB	Granzyme B	x					
ENSG00000145649	GZMA	Granzyme A	x					
ENSG00000180644	PRF1	Perforin 1	x					
ENSG00000115523	GNLY	Granulysin	x					
ENSG00000100450	GZMH	Granzyme H	x					
ENSG00000113088	GZMK	Granzyme K	x					
ENSG00000057657	PRDM1	Blimp-1	x					
ENSG00000073861	TBX21	T-bet	x					
ENSG00000115738	ID2	ID2	x					
ENSG00000176083	ZNF683	Hobit	x					
ENSG00000137265	IRF4	Interferon regulatory factor 4	x					
ENSG00000140968	IRF8	Interferon regulatory factor 8	x					
ENSG00000163508	EOMES	Eomesodermin	x			x		
ENSG00000275302	CCL4	CCL4; MIP-1alpha						x
ENSG00000139193	CD27	CD27			x			
ENSG00000178562	CD28	CD28			x			
ENSG00000126353	CCR7	CCR7						x
ENSG00000121807	CCR2	CCR2						x
ENSG00000160791	CCR5	CCR5						x
ENSG00000171791	BCL2	Bcl2				x		

ENSG00000081059	TCF7	Tcf1				x		
ENSG00000182866	LCK	Lck		x	x			
ENSG00000115085	ZAP70	ZAP70		x				
ENSG00000213658	LAT	Linker for activation of T cells		x				
ENSG00000010810	FYN	Fyn		x				
ENSG00000107968	MAP3K8	GrB2		x				
ENSG00000006062	MAPK14	MAPK14		x				
ENSG00000177885	GRB2	Growth factor receptor bound protein 2		x	x			
ENSG00000082701	GSK3B	Glycogen synthase kinase 3 beta		x	x			
ENSG00000113263	ITK	Itk		x	x			
ENSG00000170345	FOS	Fos		x				
ENSG00000177606	JUN	Jun		x				
ENSG00000171223	JUNB	JunB		x				
ENSG00000133703	KRAS	Kras		x				
ENSG00000067560	RHOA	Ras homolog family member A		x				
ENSG00000131196	NFATC1	NFATC1		x				
ENSG00000101096	NFATC2	NFATC2		x				
ENSG00000072736	NFATC3	NFATC3		x				
ENSG00000100968	NFATC4	NFATC4		x				
ENSG00000109320	NFKB1	NFKB1		x				
ENSG00000100906	NFKBIA	NFKB inhibitor alpha		x				
ENSG00000104825	NFKBIB	NFKB inhibitor beta		x				

ENSG00000146232	NFKBIE	NFKB inhibitor epsilon		x				
ENSG00000070831	CDC42	Cell division control protein 42 homolog		x			x	
ENSG00000167286	CD3D	CD3 delta chain		x				
ENSG00000198851	CD3E	CD3 epsilon chain		x				
ENSG00000160654	CD3G	CD3 gamma chain		x				
ENSG00000121879	PIK3CA	PI3K alpha subunit		x	x			
ENSG00000051382	PIK3CB	PI3K beta subunit		x				
ENSG00000171608	PIK3CD	PI3K delta subunit		x				
ENSG00000105851	PIK3CG	PI3K gamma subunit		x				
ENSG00000149269	PAK1	PAK-1		x				
ENSG00000077264	PAK3	PAK-3		x				
ENSG00000137843	PAK6	PAK-6		x				
ENSG00000100030	MAPK1	MAPK1		x				
ENSG00000102882	MAPK3	MAPK3		x				
ENSG00000185386	MAPK11	MAPK11		x				
ENSG00000050748	MAPK9	MAPK9		x				
ENSG00000156711	MAPK13	MAPK13		x				
ENSG00000169032	MAP2K1	MAP2K1		x				
ENSG00000126934	MAP2K2	MAP2K2		x				
ENSG00000076984	MAP2K7	MAP2K7		x				
ENSG00000115904	SOS1	Sos-1		x				
ENSG00000100485	SOS2	Sos-2		x				
ENSG00000158092	NCK1	NCK adaptor protein 1		x				
ENSG00000071051	NCK2	NCK adaptor protein 2		x				

ENSG00000142208	AKT1	Protein kinase B		x	x			
ENSG00000105221	AKT2	Protein kinase B beta		x	x			
ENSG00000117020	AKT3	Protein kinase B gamma		x	x			
ENSG00000135446	CDK4	Cyclin-dependent kinase 4		x				
ENSG00000139318	DUSP6	Dual Specificity Phosphatase 6		x				
ENSG00000125735	TNFSF14	LIGHT			x			
ENSG00000117586	TNFSF4	OX40 receptor			x			
ENSG00000163600	ICOS	Icos			x			
ENSG00000049249	TNFRSF9	4-1BB			x			
ENSG00000157873	TNFRSF14	HVEM			x			
ENSG00000186891	TNFRSF18	GITR			x			
ENSG00000100351	GRAP2	GRB2-related adaptor protein			x			
ENSG00000145675	PIK3R1	PI3K regulatory subunit 1			x			
ENSG00000179295	PTPN11	Ptpn11			x			
ENSG00000152256	PDK1	Pyruvate dehydrogenase kinase 1			x			
ENSG00000089692	LAG3	LAG3				x		
ENSG00000122223	CD244	CD244				x		
ENSG00000163599	CTLA4	CTLA-4				x		
ENSG00000198846	TOX	Tox				x		
ENSG00000181847	TIGIT	Tigit				x		
ENSG00000119772	DNMT3A	DNA methyltransferase 3 alpha				x		
ENSG00000153094	BCL2L11	Bcl2 like 11				x		

ENSG00000156127	BATF	Basic leucine zipper ATF-like transcription factor				x		
ENSG00000091972	CD200	CD200				x		
ENSG00000163606	CD200R1	CD200 receptor 1				x		
ENSG00000186265	BTLA	BTLA				x		
ENSG00000135077	HAVCR2	Tim-3				x		
ENSG00000141968	VAV1	Vav1					x	
ENSG00000160293	VAV2	Vav2					x	
ENSG00000134215	VAV3	Vav3					x	
ENSG00000149294	NCAM1	NCAM-1					x	
ENSG00000090339	ICAM1	ICAM-1					x	
ENSG00000131323	TRAF3	TRAF3					x	
ENSG00000111737	RAB35	Ras-related protein Rab-35					x	
ENSG00000124209	RAB22A	Ras-related protein Rab-22a					x	
ENSG00000068831	RASGRP2	Ras guanyl-releasing protein 2					x	
ENSG00000077549	CAPZB	F-actin-capping protein subunit beta					x	
ENSG00000196924	FLNA	Filamin-A					x	
ENSG00000130429	ARPC1B	Actin-related protein 2/3 complex subunit 1B					x	
ENSG00000120318	ARAP3	Arf-GAP with Rho-GAP domain, ANK repeat and PH-					x	

		domain containing protein 3						
ENSG00000184922	FMNL1	Formin-like protein 1					x	
ENSG00000070182	SPTB	Spectrin beta chain					x	
ENSG00000157827	FMNL2	Formin-like protein 2					x	
ENSG00000109920	FNBP4	Formin-binding protein 4					x	
ENSG00000220205	VAMP2	Vesicle-associated membrane protein 2					x	
ENSG00000099331	MYO9B	Myosin IXB					x	
ENSG00000006125	AP2B1	AP-2 complex sbunit beta					x	
ENSG00000130669	PAK4	PAK4					x	
ENSG00000161638	ITGA5	Integrin alpha5					x	
ENSG00000259207	ITGB3	Integrin beta-3					x	
ENSG00000005961	ITGA2B	Integrin alpha-IIb					x	
ENSG00000100345	MYH9	Myosin-9					x	
ENSG00000133026	MYH10	Myosin-10					x	
ENSG00000075624	ACTB	Beta-actin					x	
ENSG00000170458	CD14	CD14					x	
ENSG00000108622	ICAM2	ICAM-2					x	
ENSG00000163947	ARHGEF3	Rho guanine nucleotide exchange factor 3					x	
ENSG00000215375	MYL5	Myosin light chain 5					x	

ENSG00000162704	ARPC5	Actin-related protein 2/3 complex subunit 5					x	
ENSG00000136950	ARPC5L	Actin-related protein 2/3 complex subunit 5-like protein					x	
ENSG00000241553	ARPC4	Actin-related protein 2/3 complex subunit 4					x	
ENSG00000111229	ARPC3	Actin-related protein 2/3 complex subunit 3					x	
ENSG00000242498	ARPIN	Arpin					x	
ENSG00000070087	PFN2	Profilin-2					x	
ENSG00000128340	RAC2	Ras-related C3 botulinum toxin substrate 2					x	
ENSG00000129675	ARHGEF6	Rho guanine nucleotide exchange factor 6					x	
ENSG00000198752	CDC42BPB	CDC42-binding protein kinase beta					x	
ENSG00000131089	ARHGEF9	Rho guanine nucleotide exchange factor 9					x	
ENSG00000179604	CDC42EP4	CDC42 effector protein 4					x	
ENSG00000170962	PDGFD	Platelet-derived growth factor D					x	

ENSG00000174775	HRAS	GTPase Hras					x	
ENSG00000072110	ACTN1	Alpha-actinin-1					x	
ENSG00000077522	ACTN2	Alpha-actinin-2					x	
ENSG00000130402	ACTN4	Alpha-actinin-4					x	
ENSG00000172725	CORO1B	Coronin-1B					x	
ENSG00000175203	DCTN2	Dynactin subunit 2					x	
ENSG00000132912	DCTN4	Dynactin subunit 4					x	
ENSG00000104671	DCTN6	Dynactin subunit 6					x	
ENSG00000166847	DCTN5	Dynactin subunit 5					x	
ENSG00000277632	CCL3	MIP1beta						x
ENSG00000143184	XCL1	XCL1						x
ENSG00000143185	XCL2	XCL2						x
ENSG00000169245	CXCL10	CXCL10						x
ENSG00000169429	CXCL8	IL-8; CXCL8						x
ENSG00000108702	CCL1	CCL1						x
ENSG00000102970	CCL17	CCL17						x
ENSG00000275385	CCL18	CCL18						x
ENSG00000172724	CCL19	CCL19						x
ENSG00000137077	CCL21	CCL21						x
ENSG00000102962	CCL22	CCL22						x
ENSG00000274736	CCL23	CCL23						x
ENSG00000163739	CXCL1	CXCL1						x
ENSG00000138755	CXCL9	CXCL9						x
ENSG00000169248	CXCL11	CXCL11						x
ENSG00000107562	CXCL12	CXCL12						x
ENSG00000161921	CXCL16	CXCL16						x
ENSG00000121966	CXCR4	CXCR4						x

ENSG00000160683	CXCR5	CXCR5						x
ENSG00000163823	CCR1	CCR1						x
ENSG00000183625	CCR3	CCR3						x
ENSG00000183813	CCR4	CCR4						x
ENSG00000112486	CCR6	CCR6						x
ENSG00000179934	CCR8	CCR8						x
ENSG00000173585	CCR9	CCR9						x
ENSG00000184451	CCR10	CCR10						x
ENSG00000168329	CX3CR1	CX3CR1	x					x
ENSG00000172215	CXCR6	CXCR6						x
