# Supplementary material for: Functional Differences Between EBV- and CMV-Specific CD8+ T cells Demonstrate Heterogeneity of T cell Dysfunction in CLL
Source: Hemasphere. 2020 Feb 13;4(2):e337. doi: 10.1097/HS9.0000000000000337 (PMC7162091; doi:10.1097/HS9.0000000000000337)
Supplement: Supplemental Digital Content [file hs9-4-e337-s008.docx]

**Supplementary Table 2: Overview of tetramers and FACS antibodies used in this study**

| **Target molecule** | **Fluorochrome** | **Supplier** |
| --- | --- | --- |
|  |  |  |
| **EBV Tetramers** |  |  |
| HLA-A2/GLCTLVAML | APC | Sanquin Blood Supply |
| HLA-A3/RLRAEAQVK | APC | Sanquin Blood Supply |
| HLA-B7/RPPIFIRRL | APC | Sanquin Blood Supply |
| HLA-B8/RAKFKQLL | APC | Sanquin Blood Supply |
|  |  |  |
| **CMV Tetramers** |  |  |
| HLA-A2/VLEETSVML | APC | Sanquin Blood Supply |
| HLA-A2/NLVPMVATV | APC | Sanquin Blood Supply |
| HLA-B7/TPRVTGGGAM | APC | Sanquin Blood Supply |
| HLA-B8/QIKVRVDMV | APC | Sanquin Blood Supply |
| HLA-B8/ELRRKMMYM | APC | Sanquin Blood Supply |
| HLA-B8/ELKRKMIYM | APC | Sanquin Blood Supply |
|  |  |  |
| **FACS antibodies** |  |  |
| CD3 | V500 | BD |
| CD8 | BV786 | BD |
| CD45RA | BV650 | BD |
| CCR7 | BUV395 | BD |
| CD28 | PE | BD |
| CD127 | BUV737 | BD |
| PD-1 | PE-Cy7 | BD |
| CD27 | APC-eFluor780 | Thermo Fisher Scientific |
| CD244 | PE | Thermo Fisher Scientific |
| CX3CR1 | PE | Thermo Fisher Scientific |
| KLRG1 | Alexa Fluor 488 | Thermo Fisher Scientific |
| CD160 | PE-Cy7 | Biolegend |
| CXCR3 | BV711 | Biolegend |
| CD28 | FITC | Sanquin Blood Supply |
| Live/Dead Fixable Red |  | Invitrogen |
| Granzyme B | BV421 | BD |
| Granzyme K | PerCP-eFluor710 | Thermo Fisher Scientific |
| Eomes | PerCP-eFluor710 | Thermo Fisher Scientific |
| T-bet | BV421 | Biolegend |
| Ki-67 | BV711 | Biolegend |
| CD107a | FITC | Thermo Fisher Scientific |
| IFNγ | BUV395 | BD |
| IL-2 | PE | BD |
| TNFα | Alexa Fluor 700 | BD |
| MIP-1β | PE-Cy7 | BD |
