# Supplementary material for: Functional Differences Between EBV- and CMV-Specific CD8+ T cells Demonstrate Heterogeneity of T cell Dysfunction in CLL
Source: Hemasphere. 2020 Feb 13;4(2):e337. doi: 10.1097/HS9.0000000000000337 (PMC7162091; doi:10.1097/HS9.0000000000000337)
Supplement: Supplemental Digital Content [file hs9-4-e337-s009.docx]

**Supplementary Methods**

Flow cytometry analysis of CMV- and EBV-specific T cells

Peripheral blood mononuclear cells (PBMC) from CLL patients and HC were isolated and cryopreserved as described earlier^1^. To enrich for T cells, samples from CLL patients were CD19-depleted using CD19 immunomagnetic microbeads (Miltenyi Biotec, Bergish Gladbach, Germany). To identify EBV-specific T cells, PBMCs were washed with ice-cold phosphate-buffered saline containing 0.5% bovine serum albumin (PBA) and stained with EBV- or CMV-specific HLA-peptide tetrameric complexes. After tetramer labeling, PBMCs were stained with FACS antibodies for 30 minutes, followed by intracellular staining for 30 minutes using the Foxp3/Transcription Factor Staining Buffer set (Thermo Fisher Scientific). A list of used tetramers and FACS antibodies can be found in Supplemental Table 2. Cells were analyzed on an LSRFortessa flow cytometer (BD Biosciences). Data analysis was performed using Flowjo Mac Version 10.

RNA sequencing analysis

RNA sequencing analysis was carried out as described earlier^2^. Raw sequencing data were subjected to quality control using FastQC^#^, dupRadar^3^, Picard Tools^$^ and trimmed using Trimmomatic^&^ (v0.32). Reads were aligned to the human reference genome hg38 using HISAT2^£^ (v2.0.4). Gene level counts were obtained using HTSeq^§^ (v0.6.1) and the human GTF from Ensembl (release 85). Statistical analyses were performed using the edgeR^4^ and limma^5^ R/Bioconductor packages. Genes with more than 5 counts in 3 or more samples were retained. Two highly abundant mitochrondrially encoded genes (MT-RNR1 and MT-RNR2) were removed. Count data were transformed to log2-counts per million (logCPM), normalized by applying the trimmed mean of M-values method and precision weighted using voom^6^. Differential expression was assessed using an empirical Bayes moderated t-test within limma’s linear model framework including the precision weights estimated by voom and the consensus within-individiual correlation (function ‘duplicateCorrelation’, limma package). Resulting p-values were corrected for multiple testing using the Benjamini-Hochberg false discovery rate (FDR). Additional gene annotation was retrieved from Ensemble (release 92) using the biomaRt R/Bioconductor package. Multidimensional scaling analysis was performed on the logCPM values using the root-mean-square deviation (Euclidean) distance measure (function plotMDS, package edgeR). Geneset enrichment analysis was performed using CAMERA (limma package), with preset value of 0.01 for the inter-gene correlation, using a combination of all hallmark (collection H) and Biocarta and KEGG (collection C2) genesets retrieved from the Molecular Signatures Database (MSigDB v6.1; Entrez Gene ID version) and 6 manually generated genesets (Supplementary Table 3). P values were calculated for each geneset for two alternative hypotheses (‘up’ or ‘down’).

**Supplemental references**

1 Mackus, W. J. *et al.* Expansion of CMV-specific CD8+CD45RA+CD27- T cells in B-cell chronic lymphocytic leukemia. *Blood* **102**, 1057-1063, doi:10.1182/blood-2003-01-0182 (2003).

2 de Weerdt, I. *et al.* Improving CLL Vgamma9Vdelta2-T cell fitness for cellular therapy by ex vivo activation and ibrutinib. *Blood*, doi:10.1182/blood-2017-12-822569 (2018).

3 Sayols, S., Scherzinger, D. & Klein, H. J. B. B. dupRadar: a Bioconductor package for the assessment of PCR artifacts in RNA-Seq data. **17**, 428, doi:10.1186/s12859-016-1276-2 (2016).

4 Robinson, M. D., McCarthy, D. J. & Smyth, G. K. edgeR: a Bioconductor package for differential expression analysis of digital gene expression data. *Bioinformatics (Oxford, England)* **26**, 139-140, doi:10.1093/bioinformatics/btp616 (2010).

5 Ritchie, M. E. *et al.* limma powers differential expression analyses for RNA-sequencing and microarray studies. *Nucleic acids research* **43**, e47, doi:10.1093/nar/gkv007 (2015).

6 Law, C. W., Chen, Y., Shi, W. & Smyth, G. K. J. G. B. voom: precision weights unlock linear model analysis tools for RNA-seq read counts. **15**, R29, doi:10.1186/gb-2014-15-2-r29 (2014).

# <https://www.bioinformatics.babraham.ac.uk/projects/fastqc/>

$ http://broadinstitute.github.io/picard/

& <http://www.usadellab.org/cms/?page=trimmomatic>

£ <https://ccb.jhu.edu/software/hisat2/index.shtml>

§ <https://github.com/simon-anders/htseq>
